# “Who the hell is upstream pushing them all in?” Reclaiming public health’s defining metaphor to counter the commercial determinants of health

**DOI:** 10.1371/journal.pgph.0006045

**Published:** 2026-02-27

**Authors:** May C. I. van Schalkwyk, Benjamin Hawkins, Jeff Collin, Mark Petticrew

**Affiliations:** 1 Global Health Policy Unit, School of Social and Political Science, University of Edinburgh, Edinburgh, United Kingdom; 2 Centre for Pesticide Suicide Prevention, University of Edinburgh, Edinburgh, United Kingdom; 3 Addressing the Commercial Determinants of Health in Sub-Saharan Africa (ACORDS), University of Edinburgh, Edinburgh, United Kingdom; 4 MRC Epidemiology Unit, University of Cambridge, Addenbrookes Hospital, Cambridge, United Kingdom; 5 Department of Public Health, Environments and Society, London School of Hygiene & Tropical Medicine, London, United Kingdom; Institute of Public Health Bengaluru, INDIA

## Abstract

Public health policy and practice are often described by means of a metaphor that depicts interventions as “upstream” efforts to prevent people from falling into a river, from which they must be rescued “downstream” by overwhelmed healthcare services. The *upstream-downstream* metaphor has been described as public health’s defining metaphor. We apply a commercial determinants of health lens to re-engage with the initial intentions of McKinlay’s seminal 1975 essay from which this metaphor emerged, and to critique its current uses. We examine how the *upstream-downstream* metaphor has come to be used in ways that depart radically from its original intent, which was to characterise the practices of powerful commercial actors who profit from the production of harm and disease. The subtle but important shift in language from people being pushed, to falling into the river, among other depoliticising processes, contributes to an individualising and victim-blaming approach to health harms, deflecting from the role of commercial power and practices. There is a pressing need to reclaim public health’s defining metaphor as part of the wider agenda to address commercial determinants as the major public health challenges of our time.

## Background

Public health debates commonly draw on the metaphor of a river, which emerged from John B. McKinlay’s seminal 1975 article [1–3]. The article begins with McKinlay, a renowned medical sociologist and epidemiologist, [4] recounting a story – shared by his colleague Irving Zola on “the dilemmas of the modern practice of medicine” – which he uses to introduce the metaphor of *upstream* versus *downstream* factors (see Box 1) [1].

**Box 1:**

You know, he said, sometimes it feels like this. There I am standing by the shore of a swiftly flowing river and I hear the cry of a drowning man. So I jump into the river, put my arms around him, pull him to shore and apply artificial respiration. Just when he begins to breathe, there is another cry for help. So I jump into the river, reach him, pull him to shore, apply artificial respiration, and then just as he begins to breathe, another cry for help. So back in the river again, reaching, pulling, applying, breathing and then another yell. Again and again, without end, goes the sequence. You know, I am so busy jumping in, pulling them to shore, applying artificial respiration, that I have no time to see *who the hell is upstream pushing them all in* (emphasis added) [1].

The *upstream-downstream* metaphor has become ubiquitous in public health discourse, research and policy, described variously as “one of the most influential concepts in public health” [1] and as “public health’s defining metaphor” [5]. Yet the use of the metaphor has evolved away from its original use in ways that distort its meaning and power. For example, in contrast to McKinlay – who framed the problem of people being “pushed” into the river and explicitly linked this to the role of commercial actors [1] – it is now commonplace to refer to people “falling” into the river [5,6]. This subtle but important revision of the metaphor reflects a failure within the field of public health to recognise the role of powerful economic actors in creating and shaping the social structures that determine population health [7–10]. This depoliticization has been identified also in Krieger’s [9,10] critical analysis of the dominant concepts and frameworks used in the fields of public health and epidemiology. Goldberg [7] argues that concerns about the so-called politicisation of public health lead to excessively narrow, reductionist conceptualisations of the field. Similarly, Heller *et al.* [8] have identified how static and depoliticised conceptions of the social determinants of health play down the role of politics, power and vested interests in the production of health inequities at the structural level. Notably, the framework used by the World Health Organization (WHO)’s Commission on Social Determinants of Health (CSDH) differentiates between “the conditions of daily life” and “the mechanisms by which social hierarchies are created” and from which these conditions arise [11]. However, the role of the latter are often underemphasised in health policy debates that focus almost exclusively on the former [8].

The failure to recognise the political nature of these structural forces has been a key factor in the emergence and maintenance of often ineffective or sub-optimal policies which are conducive to the interests of health harming industries (HHIs) and others who benefit from the *status quo* [8,12,13]. Despite overwhelming evidence for the need to transform “upstream” (i.e., structural) determinants of ill-health and inequity to address preventable morbidity and mortality, health policy, research agendas and funding remain dominated by “downstream” interventions that emphasise individual responsibility for avoiding harms, and which place the burden of treating ill-health on health systems [3,8]. This tendency to depoliticise the major structural drivers of ill-health and inequity undermines efforts to understand and counteract them [8–10].

To cultivate more effective ways of thinking about these drivers and their resistance to change, it is essential to engage critically with the dominant ideas, norms and assumptions that underpin current public health practices and policymaking. This includes revisiting the “defining” metaphors and other rhetorical devices through which we conceptualise and explain public health issues, since metaphors shape our thinking on key policy issues, and our ability to persuade others that a particular course of action – particularly one that deviates from the historical path-dependencies of current policy approaches – is both legitimate and required [14–18].

Previous studies provide useful insights into the functions and limitations of the *upstream-downstream* metaphor, emphasising to differing degrees, the structural/ individual, proximal/ distal and temporal components of health policy problems and interventions [2,9,19–22]. However, they fail to capture fully the significance of metaphor for public health policy debates and the extent to which the *upstream-downstream* metaphor has evolved in ways which elide the key role that McKinlay identified for what he referred to as the “manufacturers of illness.” Below, we revisit McKinlay’s original article, drawing out key concepts and arguments, and provide a novel analysis of the *upstream-downstream* metaphor and its potential utility through a renewed engagement with the literature on commercial determinants of health (CDOH) and the role of metaphor in human reasoning and policymaking. In so doing, we demonstrate the importance of a more expansive (re)conceptualisation of the *upstream-downstream* metaphor for public health policy and practice.

## Metaphor and implications for public health policy

Developments in the study of metaphor provide compelling evidence for the need to critically reflect on the use of metaphors in public health research and policy discourses. Metaphors were historically regarded by some scholars and academic disciplines simply as figurative language, “clever twists of the phrase intended to add a little color to one’s wording” [23]. Their function was thus seen as distinct from literal or factual language [24]. However, as Thibodeau *et al.* [17] explain: we now know that metaphor plays a critical role in how we communicate and reason about novel, complex, and abstract subjects [17]. Lakoff and Johnson’s Conceptual Metaphor Theory identifies how “metaphor plays a very significant role in determining what is real for us” [14]. Building on this [14], subsequent research demonstrates that metaphors both *reflect* underlying conceptual representations (i.e., they *mirror* human thinking), and *shape* learning or reasoning about unfamiliar, abstract or complex issues and decisions on subsequent actions [17]. As Howarth and Griggs argue, metaphors thus provide a conceptual architecture for structuring, understanding and assigning meaning and significance to the social world [18].

Metaphors are made up of three components: a source domain (a familiar and conceptually concrete object, experience or process), a target domain (a more abstract and often unfamiliar concept or phenomena) and a conceptual mapping of particular aspects, elements or functions of the source domain onto the target domain. This conceptual process enables us to “see” or experience one thing (the target domain) in terms of another (the source domain) [15,16]. However, not all metaphors are equally potent in influencing reasoning and action. Several factors affect how influential metaphors are and previous studies have identified the affective, cognitive, social, cultural and contextual factors that enhance or curb the power of metaphor [17]. The persuasiveness of a metaphor is shaped by its emotive power or “emotional valence;” prior knowledge of the source or target domain; its positioning and extension within a given text; the extent to which it draws on and resonates with shared cultural concepts; and the audience’s prior level of interest in a source domain. Similarly, the effect of metaphor is tempered by pre-existing ideological commitments to a given issue and support for particular policy responses [17].

For example, in comparison to a neutral literal explanation, metaphorically framing the flu using emotively-charged terms like *beast, riot, army,* or *weed* that convey a powerful negative connotation increases people’s intentions to get a flu vaccination [25]. Advocating for a *war* against climate change, instead of a *race*, increases people’s sense of urgency and risk in relation to climate change and willingness to engage in more sustainable behaviours [26]. Thibodeau and Boroditsky [27] identify how metaphorically framing crime as a “virus” meant respondents were more likely to propose “treating” crime by identifying and tackling its underlying causes (i.e., through social reforms) in ways analogous with medical practice. Conversely, framing crime as a “beast” lead people to favour more confrontational measures designed to “fight back” against the aggressor and deal with the immediate threat (i.e., through augmented policing measures and more punitive sentencing for offenders) [27].

Finally, metaphors are inherently partial in that the mapping of a particular source domain on to a given target domain will inevitably foreground some aspects of the latter while others are concealed or consigned to the periphery [14,16]. Metaphors therefore privilege particular ways of thinking about problems, shaping their salience and emotive grip, and constraining the types of solutions that are seen as legitimate and necessary measures to address them [28]. The use of metaphorical framings in policy debates can been seen as acts of power in that they can help to legitimise particular framings or ways of thinking about an issue, while reproducing particular social values and norms. The metaphors invoked to explain and reason about social issues and policy problems have important implications for public health research, policymaking, and practice [29], epitomised by the *upstream-downstream* metaphor.

## Revisiting McKinlay

As described above, McKinlay’s 1975 article sets the scene for introducing the *upstream-downstream* metaphor, by recounting a story shared by his colleague in which a physician is overwhelmed trying to save an endless surge of people drowning in a fast-flowing river [1]. It is the final sentence of the parable that frames the key problem encapsulated within the metaphor; that if we want to prevent health harms, and to avoid overwhelming the healthcare services needed to treat these, we must turn our gaze “upstream” to “see who the hell is upstream pushing” people into the river and restrain their ability to do so [1]. The imperative to counter the actions of those “pushing” people into the river was not only apparent in 1975, it has intensified since. Indeed, it is now the primary challenge confronting public health. Globally, the products of just four harmful industries – tobacco, alcohol, ultra-processed food, and fossil fuels – account for at least one third of annual preventable deaths [30]. This is a considerable underestimate of the overall health impact of HHIs as it does not take into account other sectors’ products, including lead, firearms, gambling, pesticides or opioids, or industry practices such as the dumping of hazardous substances [30].

McKinlay is explicit about what he saw as the two main inferences that can be drawn from the story. First, that the health system overwhelmingly orients resources and activities towards “downstream endeavours” that fail to address the underlying drivers of ill-health [1]. Second, McKinlay argues, we must “cease our preoccupation with this short-term, problem-specific tinkering” and instead direct “our attention upstream, where the real problems lie” [1]. He explains that:

Such a reorientation would minimally involve an analysis of the means by which various individuals, interest groups, and large-scale, profit-oriented corporations are "pushing people in," and how they subsequently erect, at some point downstream, a health care structure to service the needs which they have had a hand in creating, and for which moral responsibility ought to be assumed [1].

McKinlay uses the remainder of the article to analyse the market and political activities of commercial actors and their implications for health policy and practice [1]. He achieves this by: 1) elaborating on what he calls the “manufacturers of illness,” a term used to describe individuals, interest groups and organisations who, “as an inevitable by-product” of their business activities, produce “widespread morbidly and mortality;” and 2) building the case for reorienting the focus of the public health community away from those “individuals and groups who are mistakenly held to be responsible for their condition” towards “a range of broader upstream political and economic forces” [1]. Foreshadowing later CDOH research documenting the highly consistent use of practices across HHIs [12,29,30]. McKinlay uses the food industry to illustrate “the style and magnitude of operation engaged in by the manufacturers of illness” common across sectors [1].

McKinlay explains that addressing such broad political and economic forces will involve working “upstream” to “[restrain] those who, in the interest of corporate profitability, continue to push people in” [1]. McKinlay concludes with a series of recommendations that are consistent with many of those identified decades later in CDOH literature as being key to curbing commercial practices and their negative impacts on policy and public health. Namely, he called for action in three main areas: 1) strengthening legislative interventions to curb the “pushing activities” of the manufacturers of illness; 2) efforts to address the differential power and resources between actors’ ability to lobby on health policy issues; and 3) the need to reimagine “public education” (that is, public information on health and health education programmes) to illustrate the role of commercial actors and build public outrage and support for change. Starting with the issue of legislative interventions, McKinlay states that:

It is probably true that one stroke of effective health legislation is equal to many separate health intervention endeavors and the cumulative efforts of innumerable health workers over long periods of time. […] greater changes will result from the continued politicization of illness than from the modification of specific individual behaviors [1].

In particular, he focuses on commercial advertising, proposing that placing “more stringent, enforceable restrictions on advertising” would “severely curtail the morally abhorrent pushing in activities of the manufacturers of illness”, noting the sophistication of commercial advertising and the inadequacy of existing legislation [1].

With regards to the issue of lobbying, McKinlay admits that he is at a loss as to how to address the profound differentials in resources and power at the disposal of different actors to influence health policymaking. While recognising the importance of civil society participation in the policymaking process, McKinlay highlights how, in practice, this is “a very one-sided process”, with “many legitimate interests on a range of health related issues […] structurally precluded from effective participation” in the policymaking process [1].

Finally, on the topic of public education, McKinlay described what he observed as a tendency of health messaging to focus on individual behaviour change, adopting a “blame the victim” approach, and rarely informing people about the commercial drivers of ill-health [1]. If health professionals are committed to effective health education, McKinlay posits, then they should strive to tell people the “whole story” [1]. Consequently, “immediate priority ought to be given to the sensitization of vast numbers of people to the upstream activities of the manufacturers of illness” and he argues that doing so could help to build a “groundswell of consumer interest” which may in part help to counter the “disproportionately influential lobbying of the large corporations and interest groups” [1].

In summary, the *upstream-downstream* metaphor explicitly aimed, at the very outset, to capture the practices of commercial actors, and the ways in which these are regulated, as powerful determinants of illness. It sought to re-conceptualise illness and prevention, and to reassign responsibility for ill-health [1]. In this way McKinlay captures key elements of what would later become the CDOH agenda [30].

## Critiquing the *upstream-downstream* metaphor from a CDOH perspective

As a field of research and practice, CDOH recognises the power of commercial actors as a dominant “upstream” influence on population health and equity, acting through a complex interplay of direct and indirect pathways and mechanisms [30]. The field reflects the thinking behind McKinlay’s use of the metaphor, in which countering the activities of the so-called “manufacturers of illness” or commercial actors and the conditions that enable these should be the mission and focus of the health community [1].

The utility of the *upstream-downstream* metaphor in conceptualising complex and abstract phenomena such as the social, political and economic determinants of health largely explains its emergence as public health’s defining metaphor. However, there are aspects of its usage that potentially undermine its effectiveness as a way of conceptualising CDOH and transforming policy debates and improving public health.

First, the conditions that determine health and equity, and the social and political forces that shape these conditions (the target domain), are presented and explained in terms of a natural environment (the source domain) whose treacherous conditions are being exploited for commercial gain. This imagery suggests that the existence of the river and the depth and rate of flow of the water, are all naturally occurring. Their existence, and the treacherous conditions they represent, are presented as preceding the arrival of commercial actors both upstream and further down the river. The role of commercial actors in this scene is simply to push innocent bystanders into the pre-given, natural hazard. Yet the hazards into which people are pushed – the wide array of social institutions and systems, cultural practices and spatial contexts, which shape our consumption patterns and life chances – are not naturally occurring, Rather they are the products of resource-constrained human agency and political choices that are shaped extensively by the market and political strategies of commercial actors themsleves [30]. In this regard, it is perhaps more apt to (re)conceptualise the river into which people are being actively pushed as an artificial, or man-made, waterway such as a canal. While there are instances in which the metaphor has been used to describe and explain human-driven threats to health, these often take the form of overly literal (re)interpretations of the metaphor – an upstream factory that is contaminating the water supply with toxic chemicals leading to illness in downstream communities – while the role of commercial actors and their practices, as drivers of such public health disasters, are left unexamined [22,31].

Importantly, the imagery created by the metaphor is limited in its ability to capture the health impacts of transnational corporations in the Global South, with its depiction of natural occurrence and the marginalisation of structural dynamics being particularly jarring given the history, and ongoing consequences of, colonialism [32,33]. Indeed, it risks concealing the practices of “manufacturers of illness” – often based in the Global North – as structural drivers of health harms in low- and middle-income countries, and the political and economic systems that create such structural dependencies and enable these commercial practices. The deployment of the *upstream-downstream* metaphor in low and middle income settings in its current, depoliticised form, can reflect a more general under-emphasis on the critical role played by commercial entities within the colonial project, even in progressive discourses articulating the need for decolonisation of global health [34].

The *upstream-downstream* metaphor suggests that many of the parameters in which we are working to promote health and address inequities are somehow permanent, inevitable, immutable, or outside of our control (individually and collectively). This naturalising effect of the metaphor also risks implying that countries susceptible to so-called natural disasters, notably in the Global South, are destined to experience poor health outcomes [35]. However, the very logic of the social determinants of health is that the structural forces that underpin inequities in opportunities and health are not to be seen as natural but as the outcomes of political decisions and thus can be transformed. For example, the WHO CSDH emphasised that:

This unequal distribution of health-damaging experiences is not in any sense a ‘natural’ phenomenon but is the result of a toxic combination of poor social policies and programmes, unfair economic arrangements, and bad politics [11].

Second, the *upstream-downstream* metaphor, as employed by McKinlay himself, encourages the reader to envisage commercial actors pushing people into a fast-flowing river, in broad daylight and in clear sight of the imagined observer. However, many of the most effective and harmful commercial practices are often not overtly observable and not easily conceptualised as acts of directly “pushing” people into dangerous situations. Indeed, some of the biggest effects of commercial actors on population health result from their political strategies [12] – often exercised behind closed doors and beyond public scrutiny or consciousness – that are of vital importance in shaping the policy and wider social norms and contexts in which citizens live and which determine their health outcomes.

Third, the metaphor of people being pushed into a river does not intuitively align with the types of interventions that McKinlay suggests are needed to curb the “pushing” practices of the “manufacturers of illness”: namely, restrictions on advertising, measures to redress power differentials in the policymaking space, and a re-conceptualisation of approaches to health promotion designed to stimulate a public backlash to the harmful practices of commercial actors [1]. That is, the metaphorical framing of the problem – particularly when employed without making any reference to the “manufacturer of illness” as the “pushers” – seems conceptually incongruent with the required policy solutions.

Finally, important insights can be gained from close examination of *how* the metaphor has been employed and interpreted in ways that help to depoliticise health and obscure the role of commercial actors as drivers of ill-health and inequities. For example, this has commonly been achieved by shifting the focus to “upstream” *interventions* rather than the need to resurface and debate the assumed *causal factors* and *problem definitions* [2,5]. The metaphor is often employed to describe the idea underpinning public health policy solutions and practices, as opposed to being employed to describe upstream causes including commercial actors and their practices. As described above, public health practice is now often portrayed as “going upstream” and intervening to prevent disease and promote and protect health. Yet the role of commercial actors and the politics of health and inequities have largely been downplayed or omitted within mainstream public health research and policy agendas, as well as in prominent frameworks of the structural determinants of health [36]. The terms “upstream” or “downstream” are often used in highly abstracted or ambiguous ways with little or no consideration of precisely what is happening upstream that warrants intervention, or *who* is perpetuating and profiting from these causes of harm and thus is likely to resist and undermine attempts to adopt such “upstream” measures [31,37].

## Reclaiming public health’s defining metaphor

By focusing on the provenance and functions of the *upstream-downstream* metaphor from a CDOH perspective, we demonstrate how its use within public health discourses has departed, often radically, from McKinlay’s initial intentions [5,20]. This subtle but important shift in language from people being *pushed*, to *falling* into the river, for example, contributes to an individualising and victim-blaming approach to health harms [5]. That this account of the *upstream-downstream* metaphor has come to define public health practice is highly problematic and can serve to favour the interests of commercial actors and others who benefit from the prevailing socio-economic and political order. This “metaphorical drift” points to the need for a renewed effort to reclaim the *upstream-downstream* metaphor, and to reclaim its critical edge by integrating insights from CDOH more effectively and systematically into the social determinants of health and equity agenda [36]. The importance of doing so is clearly articulated in the 2025 WHO *World report on social determinants of health equity* [38]. However, the report’s impact, and the wider CDOH agenda, risks being undermined by the same depoliticization processes documented in the wake of the CSDH [8]. It is, therefore, critical that global and public health communities and agencies continue to build and maintain an explicit commitment to recognising, researching, and addressing corporate power and practices as major structural drivers of ill-health and inequity.

Relatedly, Proctor explains that pushing “causation ‘downstream’ as far as possible” is a key element of strategies used by industries like the tobacco industry to produce ignorance about their role as structural drivers of harm [39]. This “causal truncation” functions to focus attention on certain causes of illness, located at the individual (or cellular) level by circumscribing the boundaries of legitimate academic inquiry. This leaves the *causes of causes* uninterrogated and forms part of the tobacco industry’s broader strategy of “*individuation* and *invisibilization*”, whereby the tobacco epidemic is reframed by the industry as arising from a series of disaggregated individual choices instead of as an industrial catastrophe perpetuated by corporate decisions and practices [39]. At times, public health has contributed to this strategy by adopting industry frames and terminology [39].

Those acting in the name of public health should endeavour to use the *upstream-downstream* metaphor in line with McKinlay’s initial, transformative intentions to advance health and equity, against the interests of those who profit from ill-health and inequality. A more systematic and strategic use of the evidence provided by CDOH scholarship could strengthen and build on the recommendations promoted by McKinlay and address the need he identified to curb corporate power in policymaking [40]. This requires, for example, effective systems of governance in relation to commercial engagement, following the example of Article 5.3 of the WHO Framework Convention on Tobacco Control, and counter-marketing interventions to expose and challenge industry practices [41,42].

Furthermore, public health actors should recognise the role that metaphors can play in galvanising support for policy change. They are the conceptual tools through which we make sense of complex social issues, the causes of policy problems and the legitimate policy responses to these. Simultaneously, we must guard against metaphors becoming so unreflectively embedded in policy discourses that we cease to see them as political and contestable ways of understanding the world [14].

A more critically reflective and nuanced approach to the use of metaphor, and language more generally, is key to enabling policy change within current contexts where, in conflict with the evidence base, HHIs are often seen as part of the solution to the very problems they play a core role in creating. Hence partnership working is perceived as an effective form of governance; revolving doors exist between industry, governments and regulators; conflicts of interests are concealed or tolerated; and promoting the interests of HHIs is portrayed as beneficial to wider public interests [30,43–46]. Within this paradigm, HHIs are often portrayed as important contributors to the economy, despite the negative externalities, and the substantial burden of preventable harm generated by their business practices, which threatens the sustainability of both health systems and the environment [30]. The often unreflective and uncritical approach to how the *upstream-downstream* metaphor is employed in public health discourses forecloses the possibility of stimulating potentially more effective ways of thinking and acting which may be successful in shifting the current health policy inertia.

The limitations and unintended effects of the *upstream-downstream* metaphor will need to be anticipated and addressed. Firstly, from a CDOH perspective, it is vital to emphasise that McKinlay’s metaphorical river isn’t just there waiting for hapless victims to fall or be pushed in. Instead, it is being constructed and continually reproduced and re-shaped by the very same commercial vested interests that are nudging bystanders into the fast-flowing river and overwhelming health systems. Such a perspective could be used to guide future revisions and uses of the metaphor (e.g., by referring to a canal instead of a river) to stimulate ways of rethinking, and potentially bridging, the perceived divide between differing perspectives on public health – structural versus individual, commercial versus social determinants - since it identifies commercial actors as shaping the macro-structural context through their political strategies, and individual choice architectures and preferences through product design, pricing, availability, and marketing.

Secondly, creative and effective use of metaphor and other rhetorical strategies will be needed to capture how the “manufacturers of illness” establish themselves as legitimate partners in building (pseudo)defences against their own pushing practices, such as through the strategic use of corporate social responsibility (including the creation of so-called independent organisations and charities), voluntary regulation programmes, and funding of science. Finally, promoting the use of the *upstream-downstream* metaphor as originally intended by McKinlay should not come at the cost of concealing the role of other actors, including governments, who through their own inaction, conflicts of interest or complicity, enable the harmful practices of commercial actors or employ the same practices themselves in opposition to their obligation to promote public health and equity [47].

## Conclusion

The growing interest in and recognition of CDOH provides an opportunity for the adoption of transformative and innovative approaches to public health. Reclaiming the *upstream-downstream* metaphor – public health’s defining metaphor – is a critical step to transforming and strengthening our efforts to counter the practices of the manufacturers of illness and the pathways and systems that serve their interests.
